# Astrocytic lactate shuttle disruption and the energy-starved lysosome in Alzheimer’s disease

**DOI:** 10.21203/rs.3.rs-10057949/v1

**Published:** 2026-07-15

**Authors:** YoungOuk Kim, WooMyung Heo, Se Jin Park, YoungChul Kim, Ye Eun Cho

**Affiliations:** BioXP Research Institute; BioXP Research Institute; Kangwon National University; BioXP Research Institute; Kangwon National University

**Keywords:** Alzheimer’s disease, Astrocyte-neuron lactate shuttle (ANLS), Lysosomal acidification, V-ATPase, MCT4, Cerebrospinal fluid biomarkers, Multi-omics, SEA-AD, ADNI

## Abstract

Lysosomal dysfunction is central to Alzheimer’s disease (AD), yet why structurally intact vacuolar H^+^-ATPase (V-ATPase) proton pumps fail to maintain lysosomal pH remains unresolved. Because V-ATPase activity depends on continuous ATP supply, we hypothesized that disruption of the astrocyte–neuron lactate shuttle imposes a cross-cellular energy deficit—an **“energy-starved lysosome” (ESL)** state. Integrating single-nucleus transcriptomics (SEA-AD; 1.3 million nuclei, 84 donors) with cerebrospinal fluid proteomics (ADNI Emory; n = 1,105), we found that astrocytic lactate-export genes, led by MCT4 (− 43%), declined far faster than V-ATPase, and that astrocytic MCT4 was coupled to neuronal V-ATPase independently of disease stage (donor-level partial r = + 0.466). At the protein level, V-ATPase V1A abundance was preserved across diagnostic groups—consistent with structural pump integrity—while, at the individual level, glycolytic capacity (hexokinase-1, HK1) tracked Tau pathology; this glycolysis–Tau coupling reproduced on an independent proteomic platform and against immunoassay Tau, whereas an apparent CSF V1A–Tau correlation did not survive distribution-robust analysis or validation against immunoassay Tau and is not interpreted as an individual-level marker. These findings position cross-cellular metabolic decoupling, rather than structural pump loss, as a candidate upstream constraint on lysosomal acidification, defining a candidate intervention window.

## Background

Lysosomal dysfunction is a central and early feature of Alzheimer’s disease (AD) pathogenesis, yet why structurally intact vacuolar H^+^-ATPase (V-ATPase) proton pumps fail to maintain lysosomal pH remains unresolved. Because V-ATPase activity is strictly dependent on continuous ATP supply, we reasoned that its functional failure in AD may reflect an upstream bioenergetic constraint rather than structural pump loss. Here, by integrating single-nucleus transcriptomics from the Seattle Alzheimer’s Disease Brain Cell Atlas (SEA-AD; ~1.3 million nuclei, 84 donors) with cerebrospinal fluid (CSF) proteomics from the ADNI Emory cohort (n = 1,105; 3,907 proteins), we define an ‘energy-starved lysosome’ (ESL) state in which astrocyte–neuron lactate shuttle (ANLS) genes decline sharply (MCT4 − 43%) while V-ATPase expression is preserved (− 0.8%; Δslope p = 0.0005). Astrocytic MCT4 couples with neuronal V-ATPase independently of disease progression (partial r = + 0.466), and at the protein level glycolytic capacity (HK1) tracks Tau pathology in CSF across an independent proteomic platform and against immunoassay Tau while V-ATPase V1A abundance is preserved across diagnostic groups, positioning astrocytic metabolic reserve as a cross-cellular bioenergetic constraint on lysosomal function in AD.

Autophagic–lysosomal failure leads to the accumulation of amyloid-β (Aβ) and hyperphosphorylated Tau within neurons, and impaired lysosomal acidification is now recognized as a proximal driver of both hallmark pathologies [1–4]. At the mechanistic core of lysosomal acidification sits the V-ATPase, a multi-subunit proton pump whose activity is strictly dependent on continuous ATP supply [5, 6]. Genetic evidence further implicates V-ATPase in AD: *ATP6V1A*, encoding the catalytic V1A subunit, has been identified as a top key driver of the most dysregulated neuronal gene subnetwork in late-onset AD (LOAD) across multi-omic cohorts [7], and its expression is consistently reduced in multiple brain regions from early disease stages [7, 8]. Despite this convergent evidence, a fundamental question remains unresolved: why do structurally intact V-ATPase complexes fail to maintain lysosomal pH in AD, even when pump protein is present at apparently normal abundance?

Existing mechanistic frameworks have focused on structural disruption of V-ATPase assembly. In ApoE4 carriers, cholesterol-mediated perturbation of lysosomal membrane composition impairs V-ATPase anchoring and proton translocation [9–11]. Presenilin-1 mutations disrupt lysosomal calcium homeostasis required for acidification [12]. These mechanisms, while important, are largely specific to genetic risk variants and do not fully account for the lysosomal failure observed across the broader sporadic AD population. A complementary and underexplored possibility is that V-ATPase function fails not because the pump is structurally disrupted, but because its energetic substrate—ATP—becomes insufficient. The astrocyte–neuron lactate shuttle (ANLS), in which astrocytes supply lactate as the primary oxidative fuel for neuronal ATP production [13–16], represents a candidate upstream energetic constraint whose disruption could deprive neurons of the ATP required to drive lysosomal proton pumping, even when V-ATPase protein remains structurally intact.

To test this hypothesis, we leveraged two large, independent datasets—the Seattle Alzheimer’s Disease Brain Cell Atlas (SEA-AD; ~1.3 million single nuclei, 84 donors) and the Alzheimer’s Disease Neuroimaging Initiative (ADNI) Emory TMT-MS cerebrospinal fluid (CSF) proteomics cohort (n = 1,105; 3,907 proteins)—integrating transcriptomic pseudo-progression trajectories with protein-level clinical validation. We show that astrocytic ANLS genes—most prominently MCT4/SLC16A3 (− 43%) [17]—decline far faster than V-ATPase subunit expression (− 0.8%; slope difference p = 0.0005), that this metabolic decoupling propagates across cell types and is selectively associated with markers of neuronal lysosomal dysfunction, and that independent CSF proteomic evidence confirms V-ATPase V1A protein is preserved at the group level while glycolytic capacity (HK1) tracks Tau pathology at the individual level—reproduced on an independent proteomic platform and against immunoassay Tau. Together, these data position astrocytic MCT4 decline as a candidate upstream driver of cross-cellular lysosomal energetic vulnerability—a mechanism distinct from, and complementary to, established structural routes to V-ATPase failure—and frame the MCI transition as a candidate intervention window.

## Methods

### Sex as a biological variable

The SEA-AD single-nucleus cohort (n = 84 donors) and the ADNI Emory CSF proteomics cohort (n = 1,105 subjects) include both male and female participants. Sex was included as a covariate in all weighted donor-level regression analyses (e.g., ANLS ~ CPS + age + sex) and in ANCOVA tests of CSF protein abundance across diagnostic groups (adjusting for AGE and SEX). The reported cellular and molecular phenomena are expected to apply to both sexes; no sex-specific stratification was performed because the primary findings concern population-level transcriptomic and proteomic trajectories.

### SEA-AD dataset and preprocessing

The SEA-AD single-nucleus RNA sequencing (snRNA-seq) dataset [18, 19] (SEAAD_MTG_RNAseq_final-nuclei.2024-02-13.h5ad) comprises 1,378,211 nuclei from 84 donors. Pseudo-progression bins were defined by rounding CPS to one decimal (Bins 0.1–0.9). Cell types used SEA-AD subclass annotations: astrocytes (n = 67,419), excitatory neurons (n = 671,689). Astrocyte subtypes used SEA-AD supertype annotations (Astro_1–Astro_6). Clinical metadata (Braak stage, CERAD score, NIA-AA ABC score, cognitive status, age, sex) from SEA-AD obs annotations.

### Gene modules

179 genes analyzed across lysosomal, V-ATPase (10 subunits), ANLS/glycolytic (13 genes), pH regulatory, mTOR-Ragulator, iron/redox, cholesterol, autophagy, and signaling categories. ANLS composite = mean (SLC2A1, LDHA, SLC16A1). MCT4 (SLC16A3) was analyzed separately rather than included in the composite because its trajectory was quantitatively distinct from other ANLS components (− 43.2% vs − 7.4% to − 20.8%), and its inclusion would disproportionately dominate the composite mean, obscuring the coordinated decline of the broader module. HK2 and PDK1 were excluded as they reflect glycolytic flux more broadly rather than the astrocyte-to-neuron lactate export function specifically captured by the ANLS composite. V-ATPase composite = mean of 10 subunits. LMR module: 34 genes across five functional sets.

### Statistical analysis (transcriptomic)

Bin-level means from log-normalized counts. Early = Bins 0.2–0.4; Late = Bins 0.6–0.8. Bin 0.1 was excluded from all bin-level analyses due to extreme subtype dominance (Astro_6-SEAAD 54.1% and Astro_3 31.1%, together 85.2%), which creates a leverage point that inflates correlation estimates. All bin-level statistics use Bins 0.2–0.9 (n = 8 bins; 84 donors). Pearson correlations with two-tailed p-values. Energy/Demand ratio = mean ATP-producing genes / mean V-ATPase subunits. Normalized trajectories (Bin 0.2 = 1.0) fitted with linear regression (Bins 0.2–0.9). Slope differences tested using z = (β_1_ − β_2_) / √(SE_1_^2^ + SE_2_^2^). 95% confidence intervals computed. Sensitivity analysis including Bin 0.1 (n = 9 bins) confirmed that all primary findings were robust to this exclusion: MCT4 slope remained significant (β = −0.727, p = 0.016), Δslope MCT4 vs V-ATPase remained significant (p = 0.022), and the donor-level cross-cellular coupling was unaffected (Supplemental Table 1).

### Cross-cellular analysis and partial correlations (transcriptomic)

Cross-cellular associations were computed primarily at the donor level (n = 84 donors), each donor contributing astrocyte and excitatory-neuron pseudobulk means (mean of log-normalized expression across that donor’s cells); partial correlations were computed by regressing each variable on per-donor mean CPS and correlating residuals. Bin-level correlations across nine pseudo-progression bins are reported as secondary, trajectory-level descriptors that are confounded by shared progression. Bin 0.1 was excluded from bin-level analyses due to extreme subtype dominance.

### Subtype consistency analysis

ANLS trajectories computed independently for each astrocyte supertype. Per-subtype linear regression slopes and early-to-late percentage changes computed. Subtype proportions tracked per bin.

### Network centrality

Correlation network among 28 genes (|r| > 0.7 threshold). Degree and eigenvector centrality computed.

### Clinical linkage (transcriptomic)

Donor-level aggregation was performed by Donor ID (n = 84 donors; n = 68 with CERAD). Each donor’s astrocyte and neuron values are the mean of log-normalized expression across that donor’s cells (no bin-level pre-aggregation). Spearman rank correlations were used for ordinal clinical variables (Braak, CERAD, ABC), Mann–Whitney tests for dementia status, and weighted linear regression (weight = sqrt(n_cells)) for CPS associations.

### Change-point analysis

Cumulative decline computed for each gene from Bin 0.2. Onset defined as the bin where 10% and 50% of total change occurred.

### ADNI CSF proteomics dataset

The Emory TMT-MS CSF proteomics dataset [20] was obtained from ADNI (adni.loni.usc.edu), comprising 1,170 samples and 3,907 quantified proteins (GeneName_UniProtID convention). Values of − 4 (below detection limit) were converted to NA. Clinical diagnosis (CN, MCI, Dementia) was merged from ADNIMERGE2 DXSUM [21] by RID and VISCODE; demographic covariates (AGE, SEX) from ADSL by RID. Final analytical dataset: 1,105 subjects (CN = 379, MCI = 562, DEM = 164). For orthogonal validation, CSF total tau and phosphorylated tau measured by the fully automated Roche Elecsys immunoassay [22] and an independent ADNI CSF SomaScan 7k aptamer-based proteomic matrix [23] were obtained from ADNI and matched by RID; of the SomaScan panel, MAPT, GFAP, HK1, TFRC, TREM2 and NEFL were available, whereas the V-ATPase subunits (ATP6V1A, ATP6V1E1) were not represented.

### Target protein selection and quality control

Twenty-eight target proteins were selected a priori based on the transcriptomic analysis. MCT4 (SLC16A3) was not detected, consistent with its transmembrane topology [24]. Of 28 targets, 20 had detection rates ≥ 30% and were retained. V-ATPase analyzable: V1A (n = 866, 78%) and V1E1 (n = 541, 49%).

### Diagnostic group comparison (proteomic)

Kruskal-Wallis tests assessed protein abundance differences across CN, MCI, DEM. Post-hoc pairwise Wilcoxon rank-sum tests with Holm correction. ANCOVA (Type II SS) tested diagnosis effects adjusting for AGE and SEX, with partial eta-squared (η^2^) as effect size.

### Correlation and partial correlation analysis (proteomic)

Because TMT-MS protein abundances are strongly right-skewed and Pearson correlations on untransformed values are dominated by high-abundance samples, all proteomic correlations were computed primarily by Spearman rank correlation, with Pearson on log-transformed abundance as a secondary estimate; Pearson on untransformed abundance is reported only as a sensitivity comparison. Partial correlations were computed by residualizing rank- (or log-) transformed variables on the control set by multiple regression and correlating the residuals (significance by t-test of the residual correlation). To distinguish genuine Tau association from shared per-sample/platform structure, proteomic Tau-related correlations were additionally anchored against immunoassay CSF Tau (Roche Elecsys) [22] and replicated on an independent aptamer platform (SomaScan 7k) [23]. Per-sample loading effects were assessed by median-centering log-abundances; leverage was quantified by single-subject deletion and by recomputation after trimming the top 5% of samples.

### Diagnostic group-stratified correlations

Correlations computed separately within CN, MCI, DEM. Fisher z-transformation quantified cross-group differences.

### Energy/Demand ratio (proteomic)

Each protein z-scored across all subjects. Energy composite = mean z-score (HK1, LDHA, PKM). Demand = V1A z-score. Energy − Demand compared across groups by Kruskal-Wallis.

### Software

All analyses in R v4.3.2 with hdf5r, dplyr, ggplot2, patchwork, lmerTest, car, and tidyverse.

## Results

### Coordinated ANLS decline with preserved V-ATPase

**Figure 1:**
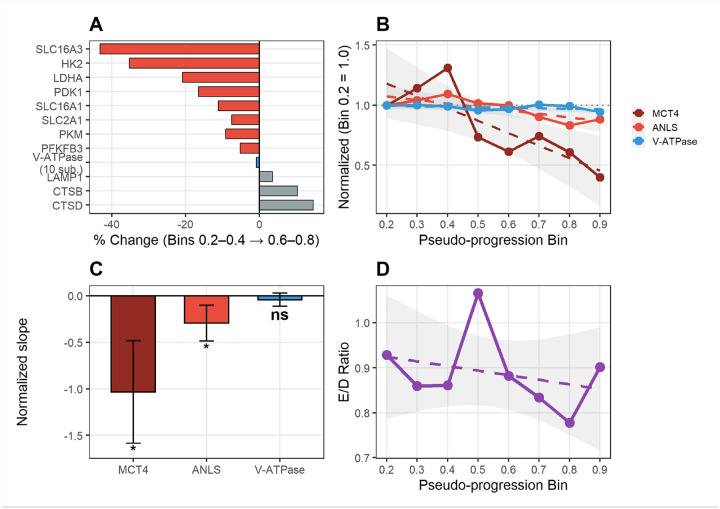
Energetic dissociation defines the energy-starved lysosome. **(A)** Percentage change in gene expression (Bins 0.2–0.4 to 0.6–0.8) for ANLS/glycolytic genes (red), V-ATPase subunits (blue), and lysosomal structural genes (grey). **(B)** Normalized trajectories (Bin 0.2 = 1.0) showing MCT4 and ANLS decline with V-ATPase stability, with 95% CI bands. **(C)** Formal slope comparison: MCT4 (β = −1.04) and ANLS (β = −0.30) decline significantly; V-ATPase slope is non-significant. Δslope MCT4 vs V-ATPase p = 0.0005. **(D)** Energy/Demand ratio trajectory declining from 0.883 to 0.831. n = 67,419 astrocytes.

We interrogated the SEA-AD single-nucleus RNA-seq atlas [18, 25] (1,378,211 nuclei from 84 donors) across nine bins of continuous pseudo-progression score (CPS) (Fig. 1A). In astrocytes (n = 67,419), ANLS/glycolytic genes [5, 13, 26, 27] showed broad, coordinated decline: MCT4/SLC16A3 (− 43.2%), HK2 (− 35.2%), LDHA (− 20.8%), PDK1 (− 16.5%), MCT1/SLC16A1 (− 11.1%), and GLUT1/SLC2A1 (− 7.4%) (Supporting Data Values). In contrast, all 10 V-ATPase subunits [5] were virtually unchanged (composite − 0.8%), and lysosomal structural genes increased (LAMP1 + 3.6%, CTSB + 10.4%, CTSD + 14.7%) across the full spectrum of AD neuropathology [19] (Fig. 1B; Supplemental Fig. 1A).

Formal dissociation testing confirmed that the ANLS decline slope (β = −0.295, 95% CI [− 0.513, − 0.077], p = 0.016) was significantly steeper than V-ATPase (β = −0.044, CI [− 0.126, 0.039], p = 0.244; Δslope z = − 2.64, p = 0.008). MCT4 showed the most dramatic dissociation (β = −1.036; Δslope vs V-ATPase z = − 3.46, p = 0.0005) (Fig. 1C). The Energy/Demand ratio declined from 0.883 to 0.831 (− 5.9%) (Fig. 1D). These data characterize the ESL state: energy supply declining significantly faster than pump expression.

### Cross-cellular propagation to neuronal lysosomes

**Figure 2:**
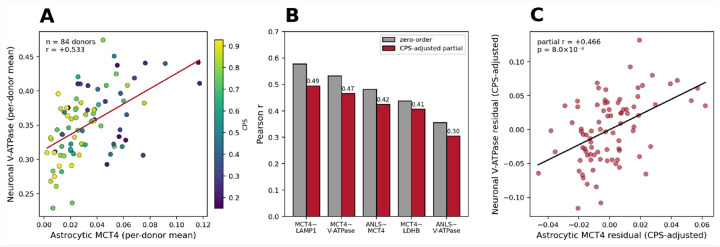
Cross-cellular metabolic coupling at the donor level. **(A)** Astrocytic MCT4 versus neuronal V-ATPase across 84 donors (Pearson r = +0.533; color = CPS). **(B)** Zero-order versus CPS-adjusted partial correlations for all five cross-cellular pairs; MCT4 couples with neuronal LAMP1 (partial r = +0.495), V-ATPase (+0.466) and LDHB (+0.407), all persisting after CPS adjustment. **(C)** Donor-level partial correlation of astrocytic MCT4 with neuronal V-ATPase after adjustment for disease progression (partial r = +0.466). n = 84 donors.

To test whether astrocytic metabolic decline propagates to neurons, we performed cross-cellular correlation analysis at the donor level (n = 84 donors; Fig. 2A). Astrocytic MCT4 was associated with neuronal V-ATPase (Pearson r = + 0.533, p = 1.9 × 10^−7^), neuronal LAMP1 (r = + 0.578, p = 8.8 × 10^−9^), and neuronal LDHB (r = + 0.438, p = 3.1 × 10^−5^)—the enzyme that oxidizes astrocyte-derived lactate for neuronal ATP production [13, 28]—defining a coherent lactate-to-lysosome supply chain (Fig. 2B). Bin-level trajectory correlations were directionally concordant but, computed across nine pseudo-progression bins, were inflated by shared progression and are reported as secondary (Supplemental Table 1).

Critically, these couplings were not merely artifacts of shared disease progression. At the donor level, partial correlation controlling for CPS confirmed that all three persisted: MCT4–neuronal V-ATPase (partial r = + 0.466, p = 8.0 × 10^−6^), MCT4–LAMP1 (partial r = + 0.495, p = 1.7 × 10^−6^), and MCT4–LDHB (partial r = + 0.407, p = 1.2 × 10^−4^), each with only modest attenuation from its zero-order value. The broader ANLS composite showed weaker coupling to neuronal V-ATPase (zero-order r = + 0.356; partial r = + 0.305, p = 4.8 × 10^−3^), whereas ANLS–MCT4 within astrocytes was stronger (r = + 0.482; partial r = + 0.425), indicating that MCT4 is the primary cross-cellular mediator rather than the broader ANLS module (Fig. 2, B and C; Table 1).

Neuronal V-ATPase declined − 5.4%—seven-fold greater than astrocytic V-ATPase (− 0.8%) (Supporting Data Values)—consistent with selective neuronal vulnerability [29] and with neurons being the primary downstream victims of astrocytic energy decline [13, 26]. Of note, neuronal MCT2 (SLC16A7)—the high-affinity lactate importer on the receiving end of the ANLS—was preserved across pseudo-progression (+ 2.3%), indicating that the neuronal lactate uptake machinery remains intact; the bottleneck is astrocytic export (MCT4 − 43.2%), not neuronal import capacity. These cross-cellular correlations support a two-layer model: ANLS disruption deprives astrocytic lysosomes of ATP directly (Layer 1), and simultaneously reduces lactate delivery to neurons, depriving neuronal lysosomes of their primary oxidative fuel [5, 13, 27] (Layer 2).

### Iron dysregulation coordinated with ANLS decline

ANLS decline was not isolated to the lactate export machinery. Iron pathway genes showed directionally consistent co-decline: TFRC (− 11.5%, r = + 0.666 with ANLS), FTH1 (− 19.2%, r = + 0.766, p = 0.027), FTL (− 15.8%), and CP (− 44.7%) [30–32] (Supplemental Fig. 1, B and C). Mitochondrial–lysosomal contact and lysosomal mTOR-activating Ragulator subunits declined in parallel (VDAC1 − 10.3%, r = + 0.761 with ANLS; Ragulator 4/5 subunits down, LAMTOR1 − 13.7%, LAMTOR2 − 17.7%; Supplemental Fig. 1D) [33–36]. SLC9A6/NHE6 [37] increased + 12.2%, peaking at Bin 0.6 — coinciding with the astrocytic compensatory peak of PTGDS, EAAT2, and ATP1A2 within the metabolic transition zone (Supplemental Fig. 1E, Supplemental Fig. 2C). This transition zone aligns temporally with the PTGDS inflection at CPS ~ 0.47 reported in our companion study [38], consistent with compensatory pH regulation during metabolic stress. Change-point analysis confirmed that MCT4 decline onset (10% at Bin 0.3) preceded iron-gene co-decline (Bin 0.5) (Supplemental Fig. 2A), while decline in the **lysosomal metabolic reserve (LMR)** composite—a 34-gene module we defined to quantify energy-dependent lysosomal function (acidification, trafficking, and substrate degradation)—preceded the iron-gene change-point by one pseudo-progression bin (Supplemental Fig. 2B).

### Subtype consistency and network architecture

**Figure 3:**
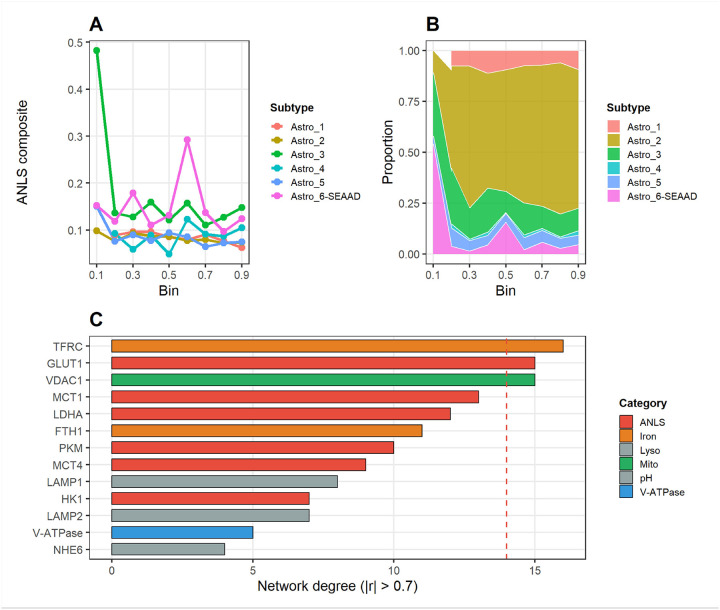
Subtype consistency and network architecture. **(A)** ANLS trajectory by astrocyte subtype: 4/6 subtypes (93.5%) show decline. **(B)**Subtype proportion shift; both expanding and contracting subtypes show within-subtype ANLS decline. **(C)** Network degree centrality: ANLS genes, TFRC, and VDAC1 as top hubs. Dashed line = 80th percentile. n = 28 genes, 128 edges.

Independent analysis across all six astrocyte supertypes [39–42] confirmed that ANLS decline occurs within 4 of 6 subtypes representing 93.5% of astrocytes: Astro_1 (− 12.5%, p = 0.021), Astro_2 (− 10.9%, p = 0.026), Astro_3 (− 6.9%), and Astro_5 (− 9.0%) (Fig. 3A). V-ATPase was preserved across all declining subtypes (− 0.2% to − 1.3%). Subtype composition shifted along pseudo-progression (Astro_2: 58.8% → 70.4%), but because ANLS declined within both expanding and contracting subtypes independently, this shift cannot account for the overall trend (Fig. 3B).

Network centrality analysis of 28 genes (|r| > 0.7, 128 edges) revealed that ANLS genes occupy the highest hub positions (GLUT1 degree = 15, 84th percentile), with TFRC (degree = 16) and VDAC1 (degree = 15) as co-hubs (Fig. 3C), consistent with the mitochondrial cascade hypothesis [43] and providing a structural explanation for why ANLS perturbation propagates broadly across the network.

### Clinical validation at the donor level

**Figure 4:**
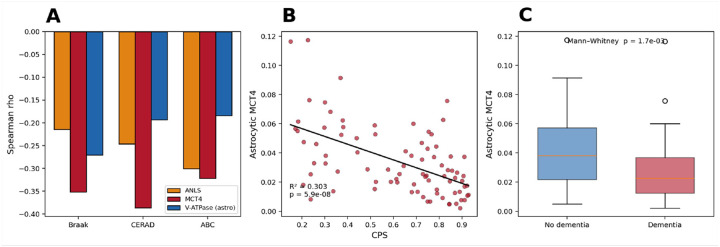
Donor-level clinical validation (true donor-level, n = 84). **(A)** Spearman correlations of ANLS, MCT4, and neuronal V-ATPase with Braak, CERAD, and ABC staging; MCT4 is strongest and most consistent, V-ATPase weakest for CERAD and ABC (consistent with its transcriptional preservation). **(B)** MCT4 declines with CPS (R^2^ = 0.303, p = 5.9×10^−8^). **(C)** MCT4 discriminates dementia status (Mann–Whitney p = 1.7×10^−3^). n = 84 donors.

Donor-level analysis (n = 84, each donor contributing its own value) confirmed that astrocytic metabolic markers—most consistently MCT4—track established pathological staging more strongly than the V-ATPase pump. MCT4 correlated with Braak stage (rho = − 0.352, p = 1.0 × 10^−3^), CERAD score (rho = − 0.386, p = 2.8 × 10^−4^), and ABC score (rho = − 0.322, p = 2.8 × 10^−3^), and discriminated dementia status (Mann–Whitney p = 1.7 × 10^−3^). ANLS showed weaker but directionally consistent associations (Braak rho = − 0.215, p = 0.05; CERAD rho = − 0.246, p = 0.024; ABC rho = − 0.300, p = 5.5 × 10^−3^) and did not discriminate dementia (p = 0.98). Astrocytic V-ATPase was consistently more weakly associated than MCT4 (Braak rho = − 0.271; CERAD rho = − 0.193; ABC rho = − 0.184) and did not discriminate dementia (p = 0.33), consistent with its relative preservation in the ESL model (Fig. 4).

Donor-level regression confirmed that MCT4 declines with progression (MCT4 ~ CPS slope = − 0.054, R^2^ = 0.303, p = 5.9 × 10^−8^), with weaker trends for ANLS (R^2^ = 0.064, p = 0.020) and neuronal V-ATPase (R^2^ = 0.085, p = 7.1 × 10^−3^); adjustment for age and sex did not alter the CPS effect (Fig. 4B). The donor-level MCT4–neuronal V-ATPase partial correlation (partial r = + 0.466, p = 8.0 × 10^−6^, n = 84) confirms that the coupling is robust to CPS adjustment (Fig. 2C; Table 1).

### CSF proteomic validation in an independent cohort

To test whether the transcriptomic ESL signature is reflected at the protein level, we analyzed the ADNI Emory tandem mass tag mass spectrometry (TMT-MS) CSF proteomics dataset [20, 44, 45] (n = 1,105 subjects; 3,907 proteins; cognitively normal (CN) = 379, MCI = 562, dementia (DEM) = 164), using distribution-robust statistics with validation against immunoassay Tau and an independent proteomic platform (see Methods).

### V-ATPase protein preservation supports the ESL model

**Figure 5:**
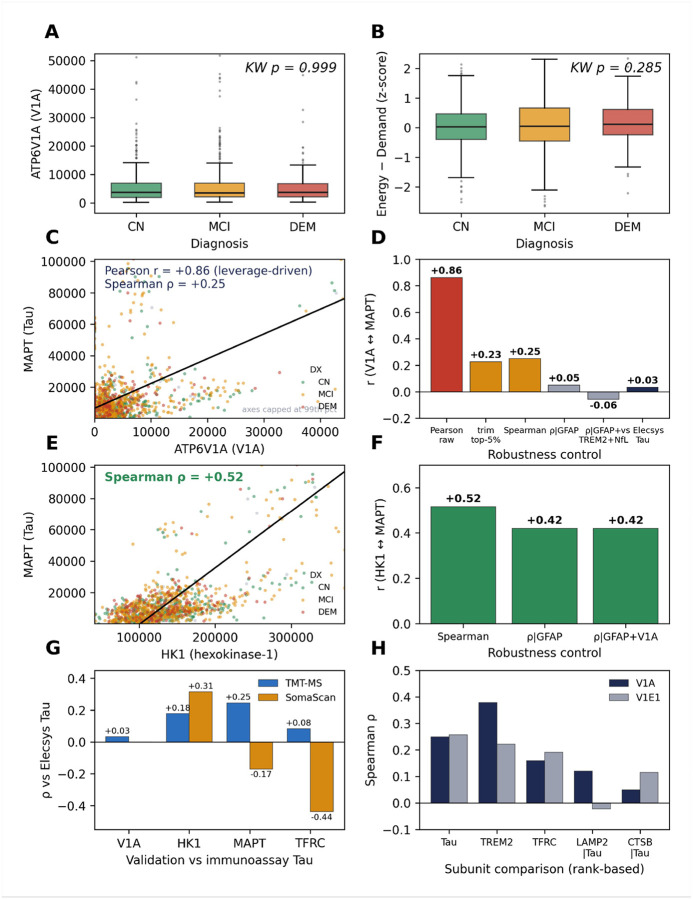
CSF proteomic validation of the energy-starved lysosome (distribution-robust). **(A)** CSF V-ATPase V1A abundance does not differ across diagnostic groups (Kruskal–Wallis p = 0.999; DEM/CN fold-change = 1.02), indicating group-level preservation. **(B)**The protein-level Energy/Demand ratio (glycolytic composite minus V-ATPase z-score) does not differ across groups (KW p = 0.285). **(C)** Untransformed CSF V1A versus Tau, illustrating a high but leverage-driven Pearson correlation (r = +0.86). **(D)** Robustness ladder for V1A–Tau: Pearson +0.86 → top-5% trimmed +0.23 → Spearman +0.25 → ρ | GFAP +0.05 → ρ | GFAP + TREM2 + NfL −0.06 → versus immunoassay (Elecsys) Tau +0.03. **(E)** HK1 versus Tau (Spearman ρ = +0.52, n = 1,170). **(F)** Robustness ladder for HK1–Tau: Spearman +0.52 → ρ | GFAP +0.42 → ρ | GFAP + V1A +0.42. **(G)** Validation against immunoassay (Elecsys) Tau on two proteomic platforms (TMT and SomaScan): HK1 is concordant on both (TMT +0.18, SomaScan +0.31), whereas V1A (+0.03) and the SomaScan MAPT (−0.17) and TFRC (−0.44) aptamers are not. **(H)** V-ATPase subunit comparison (V1A vs V1E1) for Tau, TREM2, TFRC and lysosomal markers under rank-based analysis, showing no robust subunit-specific axis. n = 1,105 (TMT); n = 1,102 (TMT–Elecsys overlap); n = 587 (SomaScan–Elecsys overlap).

Consistent with the transcriptomic finding of preserved V-ATPase mRNA (− 0.8% in astrocytes, − 5.4% in neurons), CSF V-ATPase V1A protein showed no difference across diagnostic groups (Kruskal-Wallis p = 0.999; DEM/CN fold-change = 1.018; ANCOVA with age and sex adjustment p = 0.647, η^2^ = 0.001) (Fig. 5A). This pattern extended to V1E1 (KW p = 0.308, ANCOVA p = 0.314) and glycolytic enzymes HK1 (KW p = 0.891), LDHA (p = 0.198), and PKM (p = 0.155). The protein-level Energy/Demand ratio (glycolytic composite minus V-ATPase z-score) did not differ across groups (KW p = 0.285), mirroring the modest mRNA-level decline (− 5.9%) and confirming that quantitative protein abundance is preserved despite progressive disease—consistent with the ESL model in which energetic vulnerability precedes structural loss (Fig. 5B).

By contrast, established AD biomarkers showed expected changes: CSF Tau (MAPT) was elevated in dementia (+ 24.9%, KW p = 0.003), APP was reduced (− 7.0%, KW p = 0.004), and TFRC declined (− 4.7%, KW p = 0.021) by the primary rank-based test; these group differences attenuated after age/sex adjustment (ANCOVA p = 0.15, 0.43, and 0.068, respectively)—all consistent with Tau accumulation from impaired clearance [1, 46, 47], altered APP processing [48], and iron dysregulation [30, 31], respectively (Table 2).

### Glycolytic capacity, not V-ATPase release, tracks Tau at the individual level

At the individual level, an untransformed Pearson correlation between CSF V1A and Tau was high (r = + 0.86), but these abundances are strongly right-skewed and the association was driven by a small number of high-abundance samples: trimming the top 5% of V1A values reduced the Pearson correlation from + 0.86 to + 0.23, and the distribution-robust (Spearman) estimate was + 0.25. The association did not survive control for astrocyte reactivity or axonal injury (ρ | GFAP = + 0.05; ρ | GFAP + TREM2 + NfL = − 0.06) (Fig. 5C, D). Decisively, when anchored against immunoassay CSF Tau (Roche Elecsys) rather than proteomic MAPT, V1A showed essentially no association (Spearman ρ = +0.03, n = 1,102). We therefore do not interpret CSF V1A as an individual-level Tau marker; the raw correlation reflects shared per-sample abundance structure rather than specific co-regulation.

By contrast, hexokinase-1 (HK1) tracked Tau significantly, though with modest effect size at the individual level. The HK1–Tau association persisted under rank-based analysis (Spearman ρ = +0.52, n = 1,170) and after control for astrocyte reactivity and V-ATPase abundance (ρ | GFAP = + 0.42; ρ | GFAP + V1A = + 0.42) (Fig. 5E, F). Crucially, HK1 was validated against immunoassay CSF Tau on two independent proteomic platforms: TMT-HK1 versus Elecsys Tau ρ = +0.18 (n = 1,102) and SomaScan-HK1 versus Elecsys Tau ρ = +0.31 (n = 587), whereas the SomaScan MAPT aptamer was discordant with immunoassay Tau (ρ = −0.17), underscoring the value of orthogonal validation (Fig. 5G) [22, 23]. Glycolytic capacity therefore tracks Tau pathology at the protein level across platforms, consistent with the transcriptomic ESL signature of compromised energy supply.

### Diagnostic-stage analysis

The sharp MCI-stage peak in V1A–Tau coupling reported under untransformed analysis reflects the same distribution leverage: because rank-based analysis already reduces the overall V1A–Tau association to ρ = +0.25, and to approximately zero after confound control and against immunoassay Tau, a discrete MCI-specific coupling peak is not supported. We therefore do not interpret a stage-specific V1A–Tau peak.

### Subunit-resolved analysis at the protein level (exploratory)

The two V-ATPase subunits assayed showed broadly similar associations under rank-based analysis, in contrast to their divergent untransformed correlations (Fig. 5H; Table 2).

After Tau control, the inverse associations of V1A with lysosomal markers seen in untransformed data (LAMP2, CTSB) did not persist under rank-based analysis (partial ρ | MAPT = + 0.12 and + 0.05, respectively) and are not interpreted further.

These findings are contextualized by independent experimental evidence: Wang et al. (2021) showed that CRISPRi-mediated ATP6V1A reduction in hiPSC neurons impaired electrical activity without altering lysosomal acidity [7], while Esposito et al. (2024) demonstrated that Atp6v1a depletion in murine hippocampal neurons impaired lysosomal pH and autophagy flux, with aberrant lysosome accumulation at neuronal soma and synaptic boutons [49]. The apparent discrepancy likely reflects context-dependence of energetic stress: our ESL model predicts that structurally intact V1A becomes functionally insufficient when ATP supply is limiting—a state distinct from V1A protein loss yet converging on impaired lysosomal function. Falace et al. (2024) further identify ATP6V1A as a top regulator of dysregulated neuronal subnetworks in LOAD, consistent with the selective neuronal V-ATPase decline (− 5.4%) observed here [8].

Under rank-based analysis the subunit-specific ‘axes’ suggested by untransformed correlations were not reproduced: V1A and V1E1 showed comparable associations with Tau (ρ = +0.25 and + 0.26), and V1A was, if anything, more strongly associated with the microglial marker TREM2 than V1E1 (ρ = +0.38 vs + 0.22), while their TFRC associations were similar (ρ = +0.16 vs + 0.19). The two subunits were moderately correlated (V1A ↔ V1E1 Spearman ρ = +0.24). We therefore do not assign distinct neuronal or microglial axes to the two subunits at the protein level (Fig. 5H).

### Iron pathway: independent contribution confirmed at protein level

**Figure 6:**
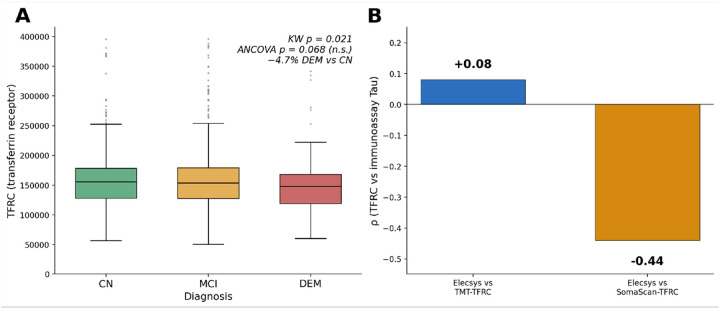
Iron pathway at the protein level. **(A)** CSF TFRC declines with disease at the group level (KW p = 0.021; non-significant trend after age/sex adjustment, ANCOVA p = 0.068), consistent with the transcriptomic TFRC–ANLS co-decline (r = +0.666) and TFRC’s network co-hub position (degree = 16). **(B)** At the individual protein level, the TFRC–Tau association was not consistent across platforms or against immunoassay (Elecsys) Tau (Elecsys vs TMT-TFRC ρ = +0.08; vs SomaScan-TFRC ρ = −0.44) and is therefore not claimed.

Transcriptomic analysis identified TFRC as a network co-hub (degree = 16) showing coordinated decline with ANLS (r = + 0.666). CSF proteomics showed group-level iron-pathway changes at the protein level: TFRC showed a group-level decline (− 4.7% DEM vs CN; KW p = 0.021)—the strongest iron-marker signal —that attenuated to a non-significant trend after age/sex adjustment (ANCOVA p = 0.068) (Fig. 6A).

At the protein level, however, the TFRC–Tau relationship was not consistent across platforms or against immunoassay Tau (Elecsys-Tau vs TMT-TFRC ρ = +0.08; vs SomaScan-TFRC ρ = −0.44) (Fig. 6B); we therefore limit the iron–Tau claim to the transcriptomic and group-level proteomic evidence and do not assert an individual-level protein TFRC–Tau coupling. CSF LCN2 did not differ significantly across diagnostic groups (KW p = 0.18; ANCOVA p = 0.11).

## Discussion

Our findings support a model in which astrocytic lactate-export collapse imposes a cross-cellular bioenergetic constraint—an ‘energy-starved lysosome’ (ESL) state—that may drive neuronal lysosomal vulnerability and is most pronounced at the mild cognitive impairment (MCI) transition, extending recent proteomic evidence of early astrocytic metabolic disruption and the mitochondrial cascade hypothesis [50, 51] to the lysosomal compartment. The formal dissociation between ANLS decline (p = 0.016) and V-ATPase preservation (p = 0.244; Δslope p = 0.0005) provides statistical evidence that energy supply declines significantly faster than pump expression. The robust association between astrocytic MCT4 and neuronal V-ATPase—which persists after CPS adjustment at the donor level (partial r = + 0.466, p = 8.0 × 10^−6^, n = 84)—suggests that this energy decline propagates across cell types via lactate supply reduction [5, 13, 27, 52]. Of note, the ANLS composite–neuronal V-ATPase association was more modest (donor-level partial r = + 0.305), indicating that MCT4 specifically—rather than the broader glycolytic module—mediates the cross-cellular relationship. Because oligodendrocytes also supply axonal lactate, this supply-line constraint may extend beyond the astrocyte–neuron axis to oligodendrocyte–axon lactate transport [53]; the present analysis focuses on astrocytic MCT4 as the primary cross-cellular mediator of neuronal lysosomal energetic vulnerability (CPS-independent coupling partial r = + 0.466).

The CSF proteomic data from an independent cohort (ADNI Emory, n = 1,105) provide protein-level corroboration of the ESL model at two points. First, V-ATPase V1A protein abundance does not differ across CN, MCI, and DEM groups (KW p = 0.999, ANCOVA p = 0.647), mirroring the transcriptomic preservation of V-ATPase mRNA (− 0.8%) and supporting the interpretation that V-ATPase protein remains available but may lack the energetic substrate required for full function. Second, glycolytic capacity (HK1) tracks Tau pathology at the individual level under distribution-robust analysis and after stringent confound control, and this coupling reproduces on an independent aptamer platform and against immunoassay Tau—providing platform-independent evidence that energy-supply capacity covaries with Tau pathology, consistent with the transcriptomic finding that MCT4–neuronal V-ATPase coupling persists after CPS control (partial r = + 0.466) and with impaired autophagic–lysosomal Tau clearance [1, 2, 46, 54, 55]. We note that an apparent individual-level CSF V1A–Tau correlation in untransformed data did not survive distribution-robust analysis or validation against immunoassay Tau, and is therefore not used to support the model; the mechanistic weight for cross-cellular V-ATPase involvement rests on the transcriptomic cross-cellular coupling and on group-level V-ATPase protein preservation.

At the protein level, the subunit-resolved analysis did not reveal a robust differential biology: although microglial lysosomal V-ATPase activity has been described [56, 57], CSF V1E1 did not show a rank-based association with microglial or iron markers that exceeded V1A (V1A–TREM2 ρ = +0.38 vs V1E1–TREM2 ρ = +0.22). We therefore present the subunit comparison as exploratory and do not interpret distinct cell-type-specific axes; this does not affect the ESL model, whose mechanistic weight rests on the transcriptomic cross-cellular coupling.

The ESL model is complementary to, rather than competing with, established structural frameworks for V-ATPase failure. Lee et al. [58] demonstrated that impaired lysosomal acidification leads to PANTHOS, a state in which Aβ accumulates within de-acidified autolysosomes [59], and attributed acidification failure to ApoE4-dependent cholesterol disruption of V-ATPase assembly [9, 10]; presenilin mutations further disrupt lysosomal calcium homeostasis required for acidification [12]. Our cross-cellular data identify a candidate upstream constraint: astrocytic MCT4 decline (− 43%) may limit lactate delivery to neurons, potentially compromising neuronal V-ATPase ATP supply even when the pump is structurally present (donor-level partial r = + 0.466). In ApoE4 carriers, both mechanisms may operate concurrently; in sporadic AD, the energetic route may provide a complementary pathway, potentially contributing to the observation that PANTHOS is not restricted to ApoE4 carriers. The Lee/Nixon framework identifies what fails (lysosomal acidification) and how neurons die [58]; our data address how energetic vulnerability may contribute to that failure despite preserved V-ATPase transcription and protein abundance (Supplemental Fig. 3). Recent multi-omic interrogation reports that V-ATPase subunit proteins are substantially reduced in late-stage AD brain tissue [59, 60] while CSF V1A remains preserved in our analysis—a discrepancy consistent with temporal staging in which CSF proteomics captures the earlier ESL phase, in which pump protein remains structurally intact but energetically compromised, whereas tissue-level reduction likely represents a later consequence of sustained energetic failure and lysosomal disassembly. This temporal-staging interpretation—CSF capturing the earlier, energetically compromised phase—is consistent with the preserved group-level V1A abundance observed here.

Our findings extend recent multi-region single-cell efforts mapping the transcriptional architecture of AD progression. Mathys et al. [25] identified an astrocyte glycolysis module (M6) with region-specific dynamics across six brain regions, peaking early in the entorhinal cortex, at intermediate stages in the hippocampus and middle temporal cortex, and late in the prefrontal cortex. This regional architecture aligns with earlier neuroimaging evidence that brain aerobic glycolysis spatially correlates with amyloid-β deposition [61], suggesting that astrocytic glycolytic capacity may delineate territories of vulnerability for downstream lysosomal–amyloid pathology. Our analysis extends this framework in three respects. First, we resolve the broader glycolytic module into its rate-limiting component, identifying MCT4 (SLC16A3) as a specific bottleneck (− 43.2%, β = −1.036) distinguishable from other glycolytic genes (HK2 − 35.2%, LDHA − 20.8%, GLUT1 − 7.4%); module-average analyses can mask such gene-level dissociations. Second, we quantitatively dissociate ANLS decline from V-ATPase preservation (MCT4 vs V-ATPase Δslope z = − 3.46, P = 0.0005), defining the energy-starved lysosome (ESL) phenotype as a structurally preserved but energetically constrained state. Third, we demonstrate cross-cellular coupling from astrocytic MCT4 to neuronal V-ATPase that persists after disease progression adjustment (donor-level partial r = + 0.466, P = 8.0 × 10^−6^), positioning ANLS disruption as a candidate proximal mediator rather than a co-occurring glycolytic phenomenon. The regional timing hierarchy described by Mathys et al. [25] also provides interpretive context for our middle temporal gyrus (MTG)-focused analysis: MTG corresponds to a region of intermediate AD vulnerability where astrocytic glycolytic compensation peaks at intermediate disease stages and transitions toward exhaustion in late stages. The MCT4 − 43% decline captured across the full pseudo-progression spectrum thus likely reflects the post-compensatory trajectory phase characteristic of intermediate-vulnerability regions. The regionally staggered timing inferred from independent multi-region data (early entorhinal, intermediate temporal, late prefrontal) predicts the same compensation → peak → decline trajectory captured at different phases per region — a hypothesis requiring within-region, donor-level validation in each region, not a claim of cross-region conservation (module-average cross-region comparisons can be confounded by global expression). Synthesizing these observations with our companion study [38], we propose a candidate multi-step cascade: astrocytic metabolic overload → PTGDS checkpoint failure and ANLS disruption → diminished lactate supply → neuronal ATP depletion → V-ATPase energetic vulnerability → impaired lysosomal acidification. Two parallel downstream consequences emerge: formation of Aβ-containing vacuoles within de-acidified autolysosomes leading to PANTHOS [58], and impaired autophagic–lysosomal clearance of hyperphosphorylated Tau.

Three additional observations contextualize this spine. First, the iron pathway contributes at the group level: CSF TFRC shows a group-level decline (KW p = 0.021) that attenuates to a non-significant trend after age/sex adjustment (ANCOVA p = 0.068), consistent with TFRC’s transcriptomic network co-hub position (degree = 16) and emerging evidence that brain iron is independently associated with cognitive decline [62, 63]; we did not, however, find a robust individual-level protein TFRC–Tau coupling across platforms. Second, the temporal architecture: in our companion study [38], astrocytic PTGDS exhibits a biphasic trajectory peaking at CPS 0.47 (quadratic vertex; raw binned maximum at Bin 0.6) before accelerated collapse, a tipping point within the metabolic transition window in which MCT4 declines. Third, the upstream trigger: the same companion study reports neuronal NDUFS1 (mitochondrial Complex I) decline as an early mitochondrial-stress feature concurrent with astrocytic PTGDS compensatory induction, consistent with ANLS failure being not a primary deficit but a consequence of unsustainable astrocytic metabolic compensation for upstream neuronal mitochondrial dysfunction. Together, these contextual observations are consistent with a cascade in which metabolic overload in astrocytes converges with disrupted lactate supply to drive neuronal lysosomal energetic vulnerability, with downstream consequences for autophagic Tau clearance and pathological Aβ accumulation within de-acidified autolysosomes (PANTHOS).

We note important limitations. SEA-AD provides cross-sectional post-mortem data; pseudo-progression infers but does not prove temporal ordering [18]. V-ATPase mRNA and protein preservation do not guarantee functional assembly [5]; lysosomal pH and ATP were not directly measured, and validation with pH-sensitive reporters [64] is needed. Bulk CSF proteomics cannot distinguish actively secreted proteins from those released by damaged cells. Importantly, CSF TMT-MS abundances are right-skewed and individual-level correlations computed by untransformed Pearson are sensitive to a few high-abundance samples [65]; we therefore used rank-based statistics as primary and validated key Tau associations against immunoassay Tau and an independent aptamer platform. Under this framework the CSF V-ATPase V1A–Tau association did not replicate and is not claimed, and cross-platform concordance for individual proteins was generally modest, underscoring that single-platform proteomic correlations require orthogonal validation. MCT4 (SLC16A3) was not detected in CSF—expected for a 12-pass transmembrane transporter—limiting direct protein-level validation of the primary ANLS hub. Independent replication and experimental validation (iPSC-derived astrocyte MCT4 knockdown with lysosomal pH measurement) and independent transcriptomic replication (e.g., ROSMAP [66]) remain essential next steps.

These data position the astrocytic lactate–neuronal V-ATPase axis as the central feature of an energy-starved lysosome state in Alzheimer’s disease: lysosomal energetic vulnerability emerges from collapse of the astrocytic lactate supply chain while V-ATPase protein remains structurally available, with cross-cellular propagation via MCT4-mediated lactate export that is most pronounced at the MCI transition in the transcriptomic data. This framework is complementary to, rather than displacing, structural models of V-ATPase failure; in ApoE4 or presenilin mutation carriers, cholesterol- and calcium-mediated assembly defects may act in parallel with the metabolic constraint identified here. At the protein level, the subunit-resolved analysis did not reveal a robust subunit-specific axis, whereas glycolytic capacity (HK1) tracks Tau across platforms; we do not interpret CSF V-ATPase subunits as individual-level markers of Tau pathology. The transcriptomic MCI-stage transition, coinciding with the PTGDS compensatory checkpoint [38], defines a window during which astrocytic metabolic support is still partially intact and intervention upstream of lysosomal failure may remain feasible. Recent independent fMRI evidence supports the clinical relevance of this window: Antal et al. [67, 68] analysed brain network instability across 19,300 individuals and identified a critical midlife transition (~ age 44) characterised by insulin-resistant glucose metabolism, with the neuronal lactate/ketone transporter MCT2 emerging as a counteracting factor and ketone supplementation in 40–59-year-olds yielding markedly greater network stabilisation than in older adults. Our cell-type-resolved analysis identifies astrocytic ANLS collapse—specifically MCT4 decline—as a candidate molecular substrate of this broader metabolic transition window, positioning astrocytic metabolic reserve as a stage-stratified therapeutic target upstream of irreversible lysosomal failure.

## Conclusions

Integrating SEA-AD single-nucleus transcriptomics (1.38 million nuclei, 84 donors) and ADNI Emory CSF proteomics (n = 1,105 subjects, 3,907 proteins), we identify cross-cellular metabolic decoupling as a candidate upstream constraint on lysosomal acidification in Alzheimer’s disease. The ‘energy-starved lysosome’ (ESL) framework explains how V-ATPase function may fail despite preserved pump expression: collapse of the astrocyte–neuron lactate shuttle (MCT4 − 43%) limits ATP supply to neuronal V-ATPase, with the strongest cross-cellular coupling at the MCI transition in the transcriptomic data. Protein-level data are consistent with this model—V-ATPase abundance is preserved while glycolytic capacity (HK1) tracks Tau across platforms—positioning astrocytic metabolic reserve as a target upstream of irreversible neuronal lysosomal failure. Future work integrating direct lysosomal pH and ATP measurement, iPSC-derived astrocyte MCT4 perturbation, and independent transcriptomic replication will be essential to advance the ESL framework from mechanistic hypothesis to therapeutic target.

## Supplementary Material

This is a list of supplementary files associated with this preprint. Click to download.

- SupportingDataValues.xlsx
- ESLGraphicalAbstract.tif
- SupplementalMaterial.docx

## Figures and Tables

**Table 1:** Cross-cellular correlations, partial correlations, and donor-level clinical associations

Measure	ANLS/MCT4	Partial r (CPS-adj)	V-ATPase	Gap
**Donor-level (n = 84)**				
MCT4 → Neuron V-ATPase	r = + 0.533***	**+ 0.466 (p = 8.0×10^−6^)**	—	—
MCT4 → Neuron LAMP1	r = + 0.578***	**+ 0.495 (p = 1.7×10^−6^)**	—	—
MCT4 → Neuron LDHB	r = + 0.438***	**+ 0.407 (p = 1.2×10^−4^)**	—	—
ANLS → Neuron V-ATPase	r = + 0.356**	**+ 0.305 (p = 4.8×10**^**−3**^)	—	—
**Donor-level clinical**				
Braak stage (rho)	−0.215 / −0.352***	—	−0.271* (astro)	gap + 0.08
ABC score (rho)	−0.300** / −0.322**	—	−0.184 (n.s.)	gap + 0.14
Dementia (Mann–Whitney p)	0.98 (n.s.) / 1.7×10^−3^	—	0.33 (n.s.)	—
**Dissociation**				
Slope (normalized)	−0.295* / −1.036*	—	−0.044 (n.s.)	Delta p = 0.0005

Note: Bin-level correlations calculated using Bins 0.2–0.9 (n = 8; Bin 0.1 excluded due to extreme subtype dominance: Astro_6-SEAAD 54.1% and Astro_3 31.1%, together comprising 85.2% of astrocytes, creating a leverage point; sensitivity analysis including Bin 0.1 confirmed robustness of all primary findings — see Supplemental Table 1). Bold partial r values survive CPS adjustment.

*** p < 0.001,

** p < 0.01,

* p < 0.05.

**Table 2:** CSF V-ATPase subunits V1A and V1E1: detection, group comparison, and distribution-robust correlations. Part A: Subunit comparison (Spearman ρ primary; raw Pearson in parentheses)

Feature	V1A (ATP6V1A)	V1E1 (ATP6V1E1)
Detection rate	866/1,105 (78%)	541/1,105 (49%)
Diagnostic group (DX) difference	KW p = 0.999	KW p = 0.308
MAPT (Tau) — Spearman ρ (raw)	+ 0.25 (+0.86)	+ 0.26 (+0.36)
vs immunoassay (Elecsys) Tau	ρ = +0.03	ρ = +0.03
TREM2 — Spearman ρ	+ 0.38	+ 0.22
TFRC — Spearman ρ	+ 0.16	+ 0.19
LAMP2 partial ρ (| MAPT)	+ 0.12	−0.02
CTSB partial ρ (| MAPT)	+ 0.05	+ 0.11
**Inter-subunit (V1A** ↔ **V1E1)**	**Spearman ρ = +0.24 (raw + 0.10)**	—
**Interpretation**	**No robust subunit-specific axis**	**(Pearson differences not preserved under rank analysis)**
**Part B: Diagnostic group changes (ANCOVA, age/sex adjusted)**		

Protein	KW p	ANCOVA DX p	eta^2	Robust?
APP	0.004	0.43	0.001	Lost
LCN2	0.181	0.11	0.004	n.s.
TFRC	0.021	0.068	0.005	Trend
MAPT	0.003	0.15	0.004	Lost
V1A	0.999	0.68	0.001	n.s.

Note: Part A partial correlations (|MAPT) represent relationship with lysosomal markers after controlling for Tau levels, indicating functional dissociation.

*** p < 0.001,

** p < 0.01,

* p < 0.05.

n.s. = non-significant.

## Data Availability

All datasets analysed in this study are publicly available from their original consortia. The Seattle Alzheimer’s Disease Brain Cell Atlas (SEA-AD) single-nucleus RNA-seq data are available at https://portal.brain-map.org/explore/seattle-alzheimers-disease and through CELLxGENE (https://cellxgene.cziscience.com/collections/1ca90a2d-2943-483d-b678-b809bf464c30). The Alzheimer’s Disease Neuroimaging Initiative (ADNI) Emory CSF TMT-MS proteomics data are available through the ADNI database (https://adni.loni.usc.edu/) upon application to the ADNI Data and Publications Committee. All analysis code supporting the findings of this study is publicly available at https://github.com/YoungOukKim/AD-Metabolic-Collapse. The repository includes both bin-level and donor-level (per-donor pseudobulk, n = 84) summary data derived from SEA-AD, sufficient to reproduce all transcriptomic figures and tables—including the donor-level cross-cellular partial correlations—without access to the raw dataset.

## Abbreviations

AD: Alzheimer’s disease
ADNI: Alzheimer’s Disease Neuroimaging Initiative
ANCOVA: Analysis of covariance
ANLS: Astrocyte-neuron lactate shuttle
APOE: Apolipoprotein E
APP: Amyloid precursor protein
ATP: Adenosine triphosphate
ATP6V1A: ATPase H+ transporting V1 subunit A (gene)
CERAD: Consortium to Establish a Registry for Alzheimer’s Disease
CN: Cognitively normal
CPS: Continuous pseudo-progression score
CSF: Cerebrospinal fluid
CTSB: Cathepsin B
DEM: Dementia
ESL: Energy-starved lysosome
GFAP: Glial fibrillary acidic protein
GLUT1: Glucose transporter 1 (SLC2A1)
HK1: HK2, Hexokinase 1, Hexokinase 2
KW: Kruskal-Wallis test
LAMP1, LAMP2: Lysosomal-associated membrane protein 1, 2
LCN2: Lipocalin-2
LDHA: Lactate dehydrogenase A
LOAD: Late-onset Alzheimer’s disease
MAPT: Microtubule-associated protein tau
MCI: Mild cognitive impairment
MCT1, MCT4: Monocarboxylate transporter 1, 4 (SLC16A1, SLC16A3)
MTG: Middle temporal gyrus
NfL: Neurofilament light chain
NIA-AA: National Institute on Aging-Alzheimer’s Association
PANTHOS: Plaque-associated neurons containing PANTHOS phenotype
PTGDS: Prostaglandin D2 synthase
SEA-AD: Seattle Alzheimer’s Disease Brain Cell Atlas
snRNA-seq: Single-nucleus RNA sequencing
TFRC: Transferrin receptor
TMT-MS: Tandem mass tag mass spectrometry
TREM2: Triggering receptor expressed on myeloid cells 2
V-ATPase: Vacuolar H+-ATPase
V1A, V1E1: V-ATPase V1 subunit A, V-ATPase V1 subunit E1
